# Complete Genome Sequence of Lacticaseibacillus paracasei Strain VHProbi F22, Isolated from Cheese

**DOI:** 10.1128/mra.00613-22

**Published:** 2022-08-31

**Authors:** Hongchang Cui, Chaoqun Guo, Zhi Duan

**Affiliations:** a Qingdao Vland Biotech Group Co., Ltd., Qingdao, China; Indiana University, Bloomington

## Abstract

Lacticaseibacillus paracasei strain VHProbi F22 is a proprietary probiotic strain that can be found in the Chinese market. Here, we investigate the whole-genome sequence of this bacterium. The whole genome contains a chromosome and a plasmid.

## ANNOUNCEMENT

A total of 1 g of cheese collected from a herdsman family in Inner Mongolia (116.2°E, 45.0°N) was serially diluted in de Man-Rogosa-Sharpe (MRS) broth medium. Then, a 10^−9^ dilution was inoculated onto an MRS agar plate and incubated at 37°C for 48 h under anaerobic conditions to enrich bacteria. The strain VHProbi F22 was classified as Lacticaseibacillus paracasei using 16S rRNA sequencing described by Liu (1). The Illumina HiSeq 2500 and the PacBio RS II single-molecule real-time (SMRT) sequencing platforms were combined to sequence the genomes of this strain. The strain was cultured under the same growth condition as those for isolation for DNA extraction. Total DNA was extracted using the genomic DNA purification kit (Promega, USA) following its instruction and was used both for the Illumina and the PacBio library preparations. Template DNA was sheared into 400 bp for the Illumina library creation using the TruSeq library preparation kit from Illumina. The sheared fragments were used to prepare the Illumina libraries using the NEXTflex rapid DNA sequencing (DNA-seq) kit (Bio Scientific, USA). The prepared libraries then were used for paired-end Illumina sequencing (2 × 150 bp). For the PacBio sequencing, DNA was spun in G-tubes (Covaris, MA) to be sheared into fragments. Then, ~10-kb fragments were purified, end repaired, and ligated with the SMRTbell sequencing adapters following the PacBio recommendation. Next, an ~10-kb insert library was prepared using the SMRTbell express template prep kit 2.0 and sequenced on one SMRT cell using standard methods. Finally, 7,024,462 raw reads and 6,801,162 clean reads were generated using the Illumina sequencing platform to reach a depth of 337.42-fold coverage, and 87,960 raw reads (*N*_50_, 12,674 bp) were generated using the PacBio sequencing platform. Adapters; non-A, -G, -C, and -T bases of the 5′ end; and reads containing >10% unknown *N* and low-quality reads were removed by using Sickle v1.33 (https://github.com/najoshi/sickle). Clean data were assembled into a scaffold using SOAPdenovo v2.04 (2). The PacBio raw reads were then assembled into a scaffold using the hierarchical genome assembly process (HGAP) and canu (3). Error correction of the PacBio assembly results was performed by Pilonjin v1.22 (4) using the Illumina reads. If there was an overlap at both ends with 5,000 bp, the loop was sequenced and one end of the loop was cut off. The final assembly generated a complete genome with a seamless chromosome and a plasmid. Glimmer v3.02 (5), tRNAscan-SE v2.0 (6), and barrnap v0.9 were used to predict protein-coding genes, tRNAs, and rRNAs, respectively (https://github.com/tseemann/barrnap/). CheckM v1.1.3 was used to check the quality of the assembled genome sequence with a completeness of 99.46%, contamination of 0%, and strain heterogeneity of 0% (7). Genome annotations were conducted using the NCBI Prokaryotic Genome Annotation Pipeline (PGAP) v5.3 (8). Default parameters were used for all software unless otherwise specified.

The whole-genome sequence of *L. paracasei* VHProbi F22 contains a 3,076,758-bp circular chromosome and a 66,795-bp circular plasmid with GC contents of 46.28%. There were 2,930 protein-coding genes, 15 rRNA genes (5S rRNA, 16S rRNA, and 23S rRNA), 61 tRNA genes, 3 noncoding RNA (ncRNA) genes, and 81 pseudogenes in the genome.

### Data availability.

The whole-genome sequence of *L. paracasei* VHProbi F22 has been deposited in the database with GenBank accession numbers CP089307 and CP089308, SRA accession numbers SRR17318357 and SRR20082622, BioProject accession number PRJNA821264, and BioSample accession number SAMN23765140.
